# Evaluation of an Automated Rapid Diagnostic Test for Detection of *Clostridium difficile*

**DOI:** 10.1371/journal.pone.0106102

**Published:** 2014-08-29

**Authors:** Masayoshi Tojo, Maki Nagamatsu, Kayoko Hayakawa, Kazuhisa Mezaki, Teruo Kirikae, Norio Ohmagari

**Affiliations:** 1 Department of Anatomy and Embryology, Faculty of Medicine, University of Tsukuba, Ibaraki, Japan; 2 Disease Control and Prevention Center, National Center for Global Health and Medicine, Tokyo, Japan; 3 Department of Infectious Diseases, Research Institute, National Center for Global Health and Medicine, Tokyo, Japan; 4 Department of Clinical Laboratory, National Center for Global Health and Medicine, Tokyo, Japan; Institute Pasteur, France

## Abstract

The Verigene *Clostridium difficile* Nucleic Acid Test (Verigene CDF Test) (Nanosphere, Northbrook, IL, USA) is a new multiplex qualitative polymerase chain reaction (PCR) test used to detect *C. difficile* toxin genes in fecal specimens. To evaluate the performance of the new method, we tested 69 fecal samples from patients with suspected *C. difficile* infection using the Verigene CDF test, an enzyme immunoassay (EIA) and PCR following anaerobic fecal culture. The sensitivity, specificity, and accuracy of the Verigene CDF test were 96.7% (29/30), 97.4% (38/39), and 97.1% (67/69) respectively, using PCR following fecal culture as a reference method. We also analyzed the potential clinical impact of the Verigene CDF test using chart reviews of the 69 patients with suspected *C. difficile* infection and found that 11 of the 69 patients were incorrectly diagnosed, and the Verigene CDF test would have led to them receiving more appropriate management including practice of treatment and contact precaution, although, of the 69 patients, there are two whose samples were incorrectly identified with the Verigene CDF test. The Verigene CDF test will have a positive impact on patient care.

## Introduction

*Clostridium difficile* (*C. difficile*) infection is a serious problem in healthcare, with high incidence, mortality, and healthcare costs [1], [2]. Accurate diagnosis is crucial to the overall management of this infection, including administration of appropriate treatment and the use of contact precautions to prevent nosocomial spread. For the diagnosis of *C. difficile* infection, various testing methods are available including enzyme immunoassays (EIAs), anaerobic fecal culture, cell culture cytotoxicity neutralization assays, and polymerase chain reaction (PCR). Although an EIA for *C. difficile* toxins A and B is easy to use and rapid, it is no longer recommended as a primary, stand-alone test due to its poor sensitivity [3], [4].

Clinical practice guidelines for *C. difficile* suggested a two-step method that uses the EIA detection of glutamate dehydrogenase (GDH) as an initial screening followed by a cell cytotoxicity assay or toxigenic culture [3], [4]. Anaerobic fecal culture is the most sensitive test and is essential for epidemiological studies, but it is not clinically practical because it does not provide timely results and has not been standardized. In Japan, a cell cytotoxicity assay, toxigenic culture, and PCR have not been introduced into routine laboratory procedures, and thus *C. difficile* infection is diagnosed mostly on the basis of EIA results and clinical symptoms.

There are now several FDA-approved assays of *C. difficile* available in the United States [5]–[12]. The Verigene *Clostridium difficile* Nucleic Acid Test (Verigene CDF Test) (Nanosphere, Northbrook, IL, USA) is a new molecular, qualitative multiplexed *in vitro* diagnostic sample-to-result test for the rapid detection of toxin A (*tcdA*) and toxin B (*tcdB*) gene sequences of toxigenic *C. difficile* from unformed (liquid or soft) fecal specimens collected from patients with suspected *C. difficile* infection. The Verigene CDF test also detects binary toxin gene sequences and the single base pair deletion at the *tcdC* gene for a presumptive identification of the epidemic *C. difficile* strain ribotype 027.

One recent study reported the comparative results regarding the performance of the Verigene CDF test with a cell culture cytotoxicity neutralization assay [12]. However, comparisons to other testing methods, such as EIAs and anaerobic fecal cultures, were not conducted, and PCR was performed only for the detection of the strain ribotype 027. The clinical impact of the Verigene CDF test was not analyzed. Here we evaluated the performance of the Verigene CDF test in comparison to those methods and its potential clinical impact, by conducting a retrospective chart review.

## Materials and Methods

### Study design and data collection

From August to October 2013 and May to June 2014, fecal samples were collected from patients with suspected *C. difficile* infection at the National Center for Global Health and Medicine (NCGM), which also serves as a tertiary hospital with 801 inpatient beds. When fecal samples were collected, all of them were tested using the EIA assay “C. Diff Quik Chek Complete” kit (TechLab, Blacksburg, VA), which detects *C. difficile* antigen (GDH) and *C. difficile* toxins (*tcdA* and/or *tcdB*).

This study included all GDH-positive and four GDH-negative samples from August 1st to October 22th 2013 (defined as the first period), and all samples submitted from May 20th to June 5th 2014 (defined as the second period), without knowledge of the patients' clinical information. A total of 400 samples were submitted during the first period. The samples analyzed during the first period were 27 GDH-positive and four GDH-negative samples, and the samples submitted and analyzed during the second period were 38 samples. The EIA-results of the 38 samples were four GDH-positive and 34 GDH-negative. The total of 69 samples were tested in the study. Of the 69 samples, 62 samples were tested within 24 hours of sample collection, and the other seven samples were tested within 4 days. All specimens were kept at 4°C until tested. A single fecal sample per patient was included in the study.

All samples were cultured anaerobically on cycloserine-cefoxitin-mannitol agar (Nissui Pharmaceutical Co., Tokyo, Japan) for the isolation of *C. difficile*. Cycloserine-cefoxitin mannitol broth with taurocholate lysozyme cysteine (CCMB-TAL broth, Anaerobe Systems, Morgan Hill, CA) for enrichment and cycloserine-cefoxitin fructose agar with horse blood and taurocholate (CCFA-HT, Anaerobe Systems) for subculture were also used during the second period. If *C. difficile* was isolated, the presence of the *tcdA*, *tcdB* and binary toxin gene was examined by PCR as described [13], [14]. PCR following the fecal culture mentioned above was performed as a reference method. The laboratory staff performing the Verigene CDF test were not aware of the results of the PCR following the fecal culture at the time of testing. Information about the administration of therapeutic antibiotics (i.e., metronidazole or oral vancomycin), the use of contact precautions and previous history of *C. difficile* infection was retrieved from patient records after these assays were performed.

### Verigene system

The Verigene system is a bench-top sample-to result platform molecular diagnostics workstation consisting of two modules: the Verigene Processor *SP* and the Verigene Reader. The user loads a specimen into the Verigene Processor *SP*, and the Verigene Processor *SP* automates the sample analysis steps including DNA extraction, PCR-based amplification, and hybridization. After the procedure, the result of the test is reported in the Verigene Reader.

### Statistical analysis

The sensitivity, specificity, and accuracy of the Verigene CDF test were examined, and the 95% confidence intervals (CIs) of them were calculated using R Software (http://www.r-project.org/). We performed a post hoc analysis of our sample size and calculated the kappa coefficient between the Verigene CDF test and the reference method, using modified R software programs [15].

### Ethical considerations

The study protocol was carefully reviewed and approved by the NCGM Ethics Committee (No. 1425). Individual informed consent was waived by the ethics committee because this study used currently existing samples collected during the course of routine medical care and did not pose any additional risks to the patients.

The tests except EIA in the study including the Verigene CDF test are not currently approved for standard clinical procedure by Japanese government and not ethically permitted for clinical diagnosis. Thus, we did not inform the clinicians of the results obtained from the methods except EIA.

## Results

### Comparison of EIA and PCR following fecal culture

Of the 31 GDH-positive samples, 13 were shown to be toxin-positive by the EIA. The PCR following fecal culture showed that, of the 31 samples, 28 were both *tcdA-* and *tcdB*-positive (Table 1). Of the 38 GDH-negative samples, there were two *tcdA-* and *tcdB*-positive samples by PCR following fecal culture (Table 1). Finally, of all the 69 samples, *tcdA-* and *tcdB*-positive and *tcdA-* and *tcdB*-negative *C. difficile* samples were 30 and 39, respectively (Table 1). As expected, the PCR following fecal culture was more sensitive than the EIA for detecting toxigenic *C. difficile* isolates.

**Table 1:** Comparison of the results of an EIA and PCR following fecal culture for detecting *C. difficile* in fecal specimens.

			PCR following fecal culture^c^	
			*tcdA+, tcdB+*	*tcdA-, tcdB-* d
EIA^a^	GDH+ (toxin+)	31 (13)^b^	28^e^ (13)^f^	3 (0)
	GDH-	38	2	36
Total		69	30	39

a The EIA detects *C. difficile* antigen (GDH) and *C. difficile* toxin (*tcdA* and/or *tcdB*).

b Of the 31 GDH-positive samples, 13 were toxin-positive in the EIA.

c According to the results of the EIA, the numbers of toxin-positive or -negative samples with PCR following fecal culture are shown.

d Numbers of samples negative with either fecal culture or PCR.

e Of the 31 GDH-positive samples, 28 were *tcdA*- and *tcdB*-positive with PCR following fecal culture.

f All 13 samples that were GDH- and toxin-positive with EIA were *tcdA*- and *tcdB*-positive with PCR following fecal culture.

### Comparison of PCR following fecal culture and the Verigene CDF test

Next, we compared the results of the Verigene CDF test with those obtained with PCR following fecal culture (i.e., the reference method). Of the 30 *tcdA-* and *tcdB*-positive samples, 29 were correctly detected with the Verigene CDF test and counted as true positive. The remaining one sample was not detected with the Verigene CDF test and counted as false negative. Similarly, Of the 39 *tcdA-* and *tcdB*-negative samples, 38 and one were recognized as true negative and false positive with the Verigene CDF test, respectively. The sensitivity, specificity, and accuracy of the Verigene CDF test were 96.7% (29/30), 97.4% (38/39), and 97.1% (67/69), respectively (Table 2). A binary toxin-positive sample was detected by the PCR following fecal culture but not by the Verigene CDF test, and this result was counted as false negative. A post hoc power analysis using our sample size showed that the statistical power (1 – type II error rate) was high enough (>80%) to generate 95% CI width of 10% for an expected accuracy 97%. The kappa coefficient was calculated to assess the diagnostic accuracy of the CDF test compared with the PCR following fecal culture as the reference method. The score was 0.94, and this result indicated almost perfect agreement between these two methods [16].

**Table 2:** Performance Characteristics of the Verigene CDF test compared with the PCR following fecal culture.

	True Positive	False Negative	True Negative	False Positive	Sensitivity (95% CI^a^)	Specificity (95% CI^a^)	Accuracy (95% CI^a^)
Verigene CDF test	29	1	38	1	96.7% (82.8–99.9)	97.4% (86.5–99.9)	97.1% (89.9–99.6)

a CI: confidence interval.

### Evaluation of clinical utility

To evaluate the potential clinical significance of the Verigene CDF test, we performed a retrospective chart review of the 69 patients whose samples were tested in the study. According to the institution's routine practices, the management of the 69 patients was determined based on the results of EIA testing and the pretest probability of *C. difficile* infection. According to the results of the Verigene CDF test and PCR following fecal culture (Table 2), 30 of the 69 patients were infected with *C. difficile* harboring *tcdA*+ *tcdB*+, and 39 patients were not. The chart reviews revealed that contact precaution was used for 20 of the 30 infected patients and for one of the 39 non-infected patients. Treatments for *C. difficile* infection (metronidazole or oral vancomycin) were administered for these 20 of the 30 infected and one of the 39 non-infected patients. The former 20 patients included all 13 toxin-positive patients by EIA shown in Table 1. The EIA-result of the latter one patient was GDH-negative and toxin-negative. Of the 30 infected patients, 10 seemed not to be recognized as having *C. difficile* infection or being a carrier, while, of the 39 non-infected patients, one seemed to be recognized as having *C. difficile* infection (Table 3). None of the former 10 patients had been diagnosed previously with a *C. difficile* infection. The 10 patients included two who had been accommodated in a private room before the EIA but were not started on contact precaution after the EIA. Whereas, there are two patients whose samples were recognized as false positive or negative with the Verigene CDF test. Both of them received appropriate management independently of the false results with the Verigene CDF test.

**Table 3:** The number of case identified or not identified as *C. difficile* infection.

	Idetntified as *C. difficile* infection	Not idetntified as *C. difficile* infection	Total
*tcdA+, tcdB+*	20^a^	10	30
*tcdA-, tcdB-*	1	38	39

a The 20 cases included 10 cases that were toxin-positive by the EIA (Table 1).

## Discussion

In this study, we evaluated the performance of the Verigene CDF test compared with other methods and we analyzed the potential clinical impact of the test by chart reviews. As reported [4], EIA was highly sensitive for detecting *C. difficile* antigen (GDH), but insensitive for detecting *C. difficile* toxins.

The Verigene CDF test showed high sensitivity, specificity, and accuracy, using the PCR following fecal culture as a reference method. A recent study showed that the concordance rates between the Verigene CDF test and a direct culture method or the Verigene CDF test and an enriched culture method were 88.4% and 92.3%, respectively [12]. The better performance of the Verigene CDF test observed in our study may be due to differences between the reference methods and/or the relatively small number of patients in the study, though the range of 95% CI in our accuracy data was less than 10% and contained the results shown in the previous report. We are now planning a large-scale prospective clinical evaluation of the Verigene CDF test.

Retrospective chart reviews revealed that 20 of the 30 infected patients and one of the 39 non-infected patients did not receive appropriate management. The exact reasons were unclear, but the patient management may have been decided based on the results of an EIA and individual situations. The management of patients with suspected *C. difficile* infection depends mainly on the results of an EIA and each physician's judgment in a country like Japan, where other testing methods are not available or are time-consuming.

Because most of the GDH-positive patients in our study had toxigenic *C. difficile* isolates, a GDH-positive but toxin-negative patient should be followed up with additional testing for the appropriate management of *C. difficile* infection, including appropriate treatment and contact precaution. While *C. difficile* infection can spontaneously resolve solely by discontinuing antibiotics [17], this patient population may include asymptomatic carriers. It is important that contact precaution is used for these patients at the optimal timing, because they serve as a potential reservoir for environmental contamination to other hospitalized patients [18].

Several methods to recover *C. difficile* from stool samples was evaluated to determine sensitive method [19]. During the second period, we also used CCMB-TAL for enrichment and CCFA-HT for subculture, which were more sensitive than CCMA in recovering *C. difficile* from stool samples. Of all samples submitted during the second period, one sample was isolated with only CCMB-TAL and CCFA-HT, not with CCMA (data not shown). During the first period, three samples of five *tcdA-* and *tcdB*-negative were culture-negative with CCMA, and we cannot deny that some of them might have been culture-positive with CCMB-TAL and CCFA-HT.

PCR following fecal culture as our reference method was time-consuming and used mainly for confirmation of the diagnosis of *C. difficile*, and thus it may be difficult to evaluate exactly the potential clinical impact of the Verigene CDF test compared to our reference method. However, the Verigene CDF test has some strengths, such as its simple and quick procedures which do not require trained laboratory personnel. This test could be an appropriate alternative to PCR for detecting *C. difficile*, especially in countries where PCR has not been introduced into routine laboratory procedures.
